# *Helminthostachys zeylanica* alleviates hepatic steatosis and insulin resistance in diet-induced obese mice

**DOI:** 10.1186/s12906-019-2782-3

**Published:** 2019-12-13

**Authors:** Ting-Chen Chang, Hao Chiang, Yu-Heng Lai, Yu-Ling Huang, Hsiu-Chen Huang, Yu-Chih Liang, Hui-Kang Liu, Cheng Huang

**Affiliations:** 1 Ph.D. Program in Medical Biotechnology, College of Medical Science and Technology, Taipei Medical University, Taipei, 11001 Taiwan; 2 Department of Biotechnology and Laboratory Science in Medicine, National Yang-Ming University, No. 155, Sec. 2, Linong St., Beitou District, Taipei, 11221 Taiwan; 3 Department of Chemistry, Chinese Culture University, Taipei, 11114 Taiwan; 4 National Research Institute of Chinese Medicine, Ministry of Health and Welfare, Taipei, 11221 Taiwan; 5 Department of Cosmetic Science, Chang Gung University of Science and Technology, Taoyuan, Taiwan; 6 Department of Applied Science, National Tsing Hua University, Hsinchu, 30014 Taiwan; 7 School of Medical Laboratory Science and Biotechnology, College of Medical Science and Technology, Taipei Medical University, Taipei, 11001 Taiwan; 8 Ph.D. Program in Clinical Drug Development of Chinese Herbal Medicine, Taipei Medical University, Taipei, 11001 Taiwan; 9 Department of Earth and Life Sciences, University of Taipei, Taipei, 11153 Taiwan

**Keywords:** *Helminthostachys zeylanica*, Hepatic steatosis, Insulin resistance, Obesity

## Abstract

**Background:**

Obesity and its associated health conditions, type 2 diabetes mellitus (T2DM) and nonalcoholic fatty liver disease (NAFLD), are worldwide health problems. It has been shown that insulin resistance is associated with increased hepatic lipid and causes hepatic steatosis through a myriad of mechanisms, including inflammatory signaling.

**Methods:**

*Helminthostachys zeylanica* (HZ) is used widely as a common herbal medicine to relieve fever symptoms and inflammatory diseases in Asia. In the present study, we evaluated whether HZ has therapeutic effects on obesity, NAFLD and insulin resistance. The protective effects of HZ extract were examined using free fatty acid-induced steatosis in human HuS-E/2 cells and a high-fat diet-induced NAFLD in mice.

**Results:**

The major components of the HZ extract are ugonins J and K, confirmed by HPLC. Incubation of human hepatocytes, HuS-E/2 cells, with palmitate markedly increased lipid accumulation and treatment with the HZ extract significantly decreased lipid deposition and facilitated AMPK and ACC activation. After 12 weeks of a high-fat diet with HZ extract treatment, the HFD mice were protected from hyperlipidemia and hyperglycemia. HZ extract prevented body weight gain, adipose tissue expansion and adipocyte hypertrophy in the HFD mice. In addition, fat accumulation was reduced in mice livers. Moreover, the insulin sensitivity-associated index, which evaluates insulin function, was also significantly restored.

**Conclusions:**

These results suggest that HZ has a promising pharmacological effect on high-fat diet-induced obesity, hepatic steatosis and insulin resistance, which may have the potential for clinical application.

## Background

Obesity, fatty liver and dysregulated insulin action are strongly associated and are currently a worldwide health problem [1]. Fatty liver is the initial stage of nonalcoholic fatty liver disease (NAFLD), which is caused by an imbalance of lipid metabolism and is a common metabolic symptom [2]. NAFLD and type 2 diabetes mellitus (T2DM) frequently coexist because of sharing the similar pathogenic features of excess adiposity and insulin resistance [3]. Dysregulation of hepatic lipid homeostasis is thought to be important in the development of fatty liver, such as reduced fatty acid oxidation, enhanced de novo lipogenesis, elevated hepatic fatty acid influx, and/or increased systemic insulin resistance [4]. Although the mechanisms involved in lipid-induced insulin resistance are not fully understood, it is well-known that nonalcoholic fatty liver disease (NAFLD), which causes nonalcoholic steatohepatitis (NASH), is one of the most relevant factors that leads to metabolic diseases and insulin resistance [5]. Indeed, therapies for fatty liver disease are aimed at reducing body weight and improving insulin sensitivity to alleviate the associated metabolic syndrome [6, 7]. Now, novel therapeutic strategies for NAFLD progression have stimulated great interest in terms of developing effective treatments for lipid-associated metabolic disorders [8].

*Helminthostachys zeylanica* (L.) Hook. (HZ), the only fern-like plant of the Ophioglossaceae, is distributed widely in Southeast Asia and has been used as a folk medicine for centuries [9]. It has been shown that HZ contains prenylated flavonoids and quercetin, which have inhibitory effects on human neutrophils [10]. In addition, the main components in HZ, flavonoids, have antioxidant and anti-inflammatory activities [10, 11]. Previous studies have shown that one of the main compounds in HZ, ugonin K, promotes osteogenesis through the Src-associated pathway and activates downstream Runx2 and oxterix [12]. Furthermore, HZ extract was considered to have neuroprotective activity because of its anti-inflammatory activity on human astrocytes, through bradykinin-induced MMP-9 signaling [13]. Another bioactive compound extracted from HZ, ugonin J, is considered to be a potential inhibitor of cell migration and neointima formation through MMP-2 and -9 pathways [14]. Rhizomes of HZ have been used for variety of purposes, including protection against liver damage [15]. However, the therapeutic effect of HZ on abnormalities of lipid and glucose metabolism remains unclear.

Previously, we established a human fatty liver cell model, based on HuS-E/2 immortalized human primary hepatocytes [16], and made use of a mouse model of metabolic syndrome with high-fat diet (HFD), which showed significant dyslipidemia and insulin resistance, and expressed hepatic steatosis markers [17]. Because of the vicious circle between NAFLD and insulin resistance, in this study, we applied our optimized human fatty liver cell model and HFD mouse model of metabolic disorder and investigated the potential restorative therapeutic effects of HZ.

## Methods

### *H. zeylanica* (HZ) extract preparation

Rhizomes of HZ were purchased from the Wanhua herbal market (Taipei, Taiwan) and identified by comparison with the voucher specimen (NRICM-99-003), which is already deposited at the herbarium of the National Research Institute of Chinese Medicine, Taiwan. HZ rhizomes (531 g) were heated and extracted with 2.5 l of EtOH-H_2_O (1:1) under reflux for 1 h. The filtrate was concentrated and lyophilized to yield HZ extract (29 g, yield 5.46%).

### Purification of ugonin J and ugonin K

The preparation of ugonins J and ugonin K were prepared as described previously [11]. Briefly, the rhizomes of HZ (12 kg) were extracted with EtOH (20 l × 3) at 50 °C for 24 h. The concentrated EtOH extract (460 g) was partitioned between EtOAc and H2O, and the EtOAc extract (153 g) was applied to a silica gel column eluted with gradient solvent systems of *n*-hexane–EtOAc (20:1–1:10) and EtOAc–MeOH (10:1–1:1) to yield 16 fractions (Fr-1–Fr-16). Fraction Fr-7, the eluate of *n*-hexane–EtOAc = 1:2, was further subjected to a silica gel CC (CH2Cl2–MeOH = 30:1) and Sephadex LH-20 (MeOH–H2O = 5:1) to give ugonin J (26.3 mg) and ugonin K (18.6 mg), respectively.

### Reverse-phase HPLC chemical fingerprint analysis of HZ extract

The HZ extract (1.0 g) was refluxed in 20 ml methanol for 30 min and filtered. The filtrate volume was then adjusted to 50 ml with the same solvent. A 10 μl portion of the solution was injected into the HPLC system, an Agilent 1100 series equipped with a G1311A Quat Pump, a G1379A degasser, a G1315B photodiode array detector, a 1200 series G1329A autosampler, and a column oven H-650 (Chrom Tech, TNC.). A Cosmosil 5C18-AR-II column was utilized with a mobile phase of MeOH-H_2_O (0.1% phosphoric acid, v/v) using a linear gradient, which started from 70% MeOH for 35 min, increasing to 75% in 10 min, and finally reaching 100% at 65 min with flow rate of 1.0 ml/min. The column oven was set at 30 °C and the UV detection wavelength was set at 344 nm.

### Antibodies, reagents and Western blot analysis

Palmitate, Oil red O, and luteolin were purchased from Sigma-Aldrich, St. Louis, MO. Antibodies against AMPK, pACC (Ser 79), ACC, SREBP-1c, CPT1, and tubulin were from Genetex. The anti-pAMPK (Thr 172) antibodies were obtained from Millipore, and the HRP-conjugated anti-mouse or anti-rabbit secondary antibodies were from Jackson ImmunoResearch Laboratories Inc. Western blot analysis was performed as described previously [16].

### Cell culture and oil red O staining

HuS-E/2 cells were kindly provided by Dr. Shimotohno (Kyoto University, Japan) and maintained as described previously [18]. The lyophilized HZ extract was solubilized in DMSO as stock at the concentration of 25 mM and diluted to the indicated concentration. DMSO was used as the vehicle for experimental control. For the fatty liver disease cell model, HuS-E/2 cells were cultured with 0.1 mM palmitate for 18 h. To measure lipid content in HuS-E/2 cells, the oil red O method was used as previously described [16].

### Quantitative real-time polymerase chain reaction

For messenger RNA (mRNA) analysis, real-time polymerase chain reaction (RT-PCR) was performed as described previously [19]. The primer sets used in this study are listed in Additional file 1: Table S1.

### Animals

4-week-old male C57BL/6 J mice were obtained from BioLASCO Taiwan Co, Ltd., Taiwan. All mice were housed under constant temperature (24 °C) with a 12 h light/dark cycle at the Animal Center of the National Research Institute of Chinese Medicine, Taipei, Taiwan. Mice fed with a standard diet and adapted to the environment for one week were subsequently divided randomly into three groups and fed a normal diet (ND group, *n* = 10), HFD (HFD group, n = 10, 30% fat and 1% cholesterol), or HFD with 0.5% HZ extract (HFD-HZ group, n = 10) for 12 weeks. The estimated daily intake amount of HZ extract is about 578 mg/kg/day. On the day of sacrifice, a laparotomy was performed under ketamine and xylazine anesthesia (intramuscular injection of 100 mg/kg body mass and 5 mg/kg body mass, respectively), and mice were sacrificed via cardiac-puncture (to collect blood for the final time point) followed by cervical dislocation. The liver and adipose tissue were removed, rinsed with physiological saline, weighed, immediately frozen in liquid nitrogen, and stored until analysis. The animals used in this research were approved by the Animal Research Committee of the NRICM (IACUC no. 105–520-2). All experimental procedures were followed The Guide for the Care and Use of Laboratory Animals (NIH publication, 85–23, revised 1996) and the guidelines of the Animal Welfare Act, Taiwan.

### Biochemical characterization of plasma and histological analysis of fat and liver tissues

The plasma, epididymis adipose, and liver tissues were collected from each sacrificed mouse. The biochemical analysis of plasma and histological analysis of the fat and liver tissues were performed as described previously [20].

### Blood glucose, plasma insulin and the homeostasis model assessment of insulin resistance index (HOMA-IR)

The 12 h fasting blood glucose was measured with a glucose analyzer (EASYTOUCH, Taiwan). The plasma insulin and HOMA-IR were detected and calculated as described previously [20].

### Statistical analysis

All data are expressed as the mean ± SD from for three separate experiments. More than two sets of data were accessed by one-way ANOVA with Dunnett’s multiple comparison test. The values significantly different from the control were indicated by asterisks (*, *p* < 0.05; **, *p* < 0.01; ***, *p* < 0.001.).

## Results

### Identification of major components in *H. zeylanica* (HZ) extract

**Fig. 1:**
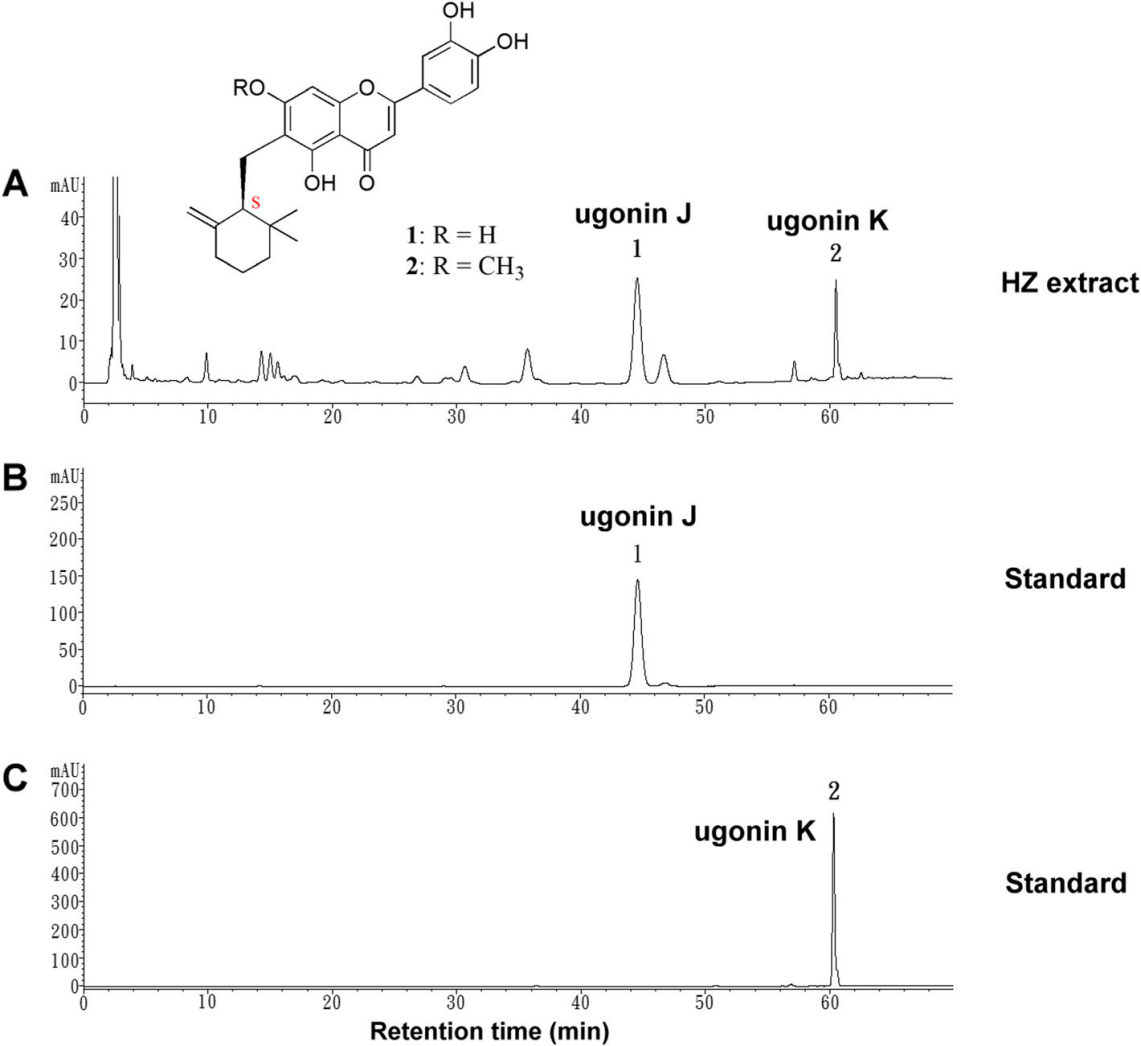
Characterization of HZ extract. **a** HPLC chromatograms of HZ extract. Two major peaks were identified in the HZ extract. **b** Ugonin J and (**c**) ugonin K were used as standards. The chemical structures of the ugonins are shown

Rhizomes of HZ were extracted and the chemical components were analyzed. HPLC analysis was performed on the HZ extract and two of the individual ingredients, ugonins J and K were isolated [11] and used as standard markers for quality control of HZ material. Both standard markers were well separated and their purities were determined by HPLC to be more than 98%. The HPLC chromatogram of the HZ extract showed two major peaks at 44.484 and 60.466 min. (Fig. 1a), corresponding to ugonin J (44.588 min.) (Fig. 1b) and ugonin K (60.276 min.) (Fig. 1c) under the same conditions.

### The effect of HZ on palmitate-induced cellular lipid accumulation in a human fatty liver cell model

**Fig. 2:**
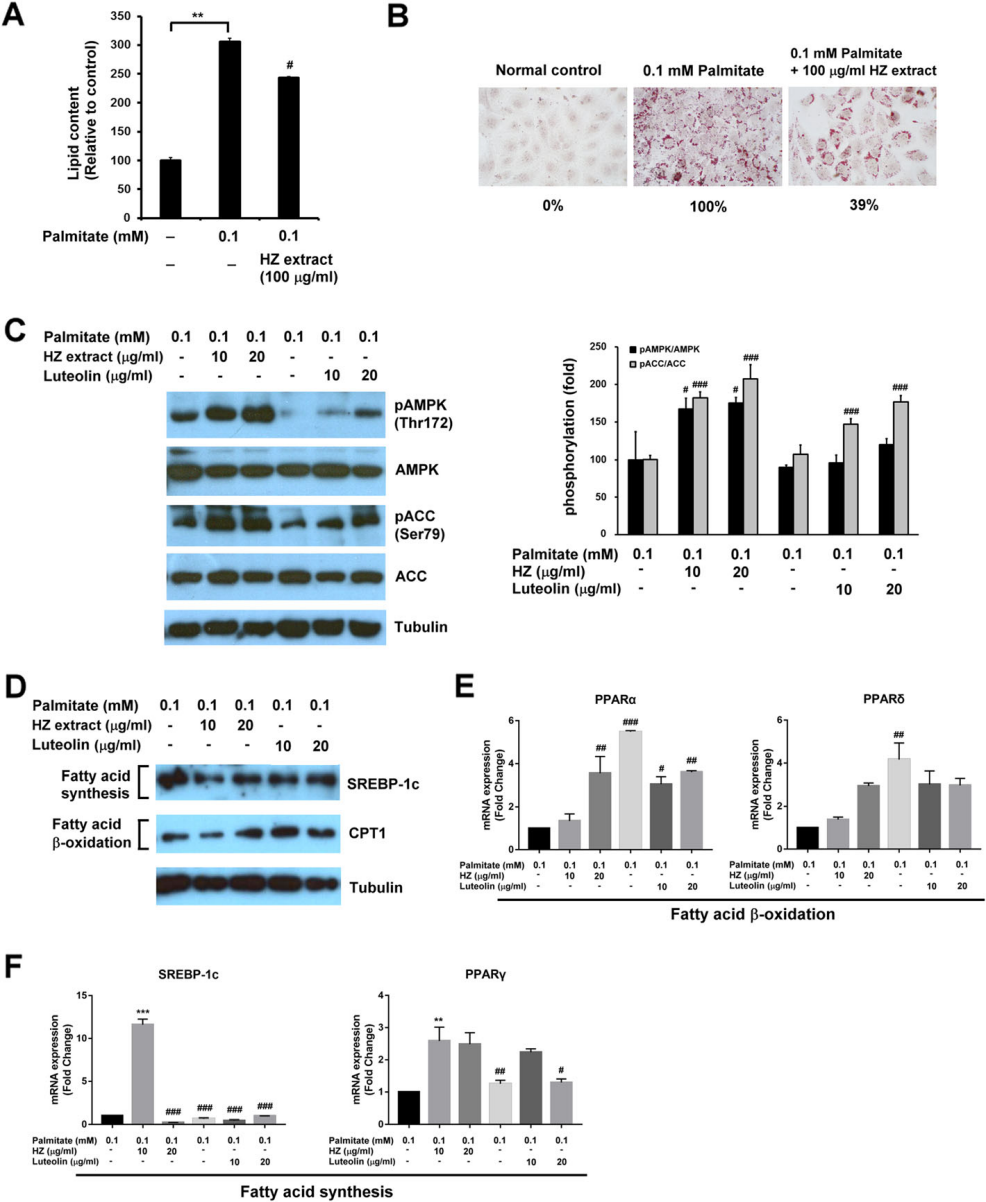
Inhibition by HZ extract of palmitate-induced lipid accumulation in a human fatty liver cell model. **a** Quantitative analysis of lipid deposition in the Oil-Red O stained HuS-E/2 cells. **b** Images of the Oil-Red O stained HuS-E/2 cells were captured using a microscope at 400X original magnification. **c** Western blotting for phosphorylation of AMPK at Thr172 and ACC at Ser-79, total AMPK, ACC and tubulin. **d** Western blotting for SREBP-1c, CPT1, and tubulin. Tubulin served as a loading control. Quantitative analysis with Multi Gauge V3.0 is shown. **e** The levels of fatty acid β oxidation-related genes, PPARα and PPARδ. **f** The levels of fatty acid synthesis-related genes, SREBP-1c and PPARγ. The data represent the mean ± SEM for three independent experiments. ND vs. palmitate: *p < 0.05; **p < 0.01; ***p < 0.001. palmitate vs. HZ extract: #*p* < 0.05; ##*p* < 0.01; ###*p* < 0.001

Fatty liver disease is mainly attributable to triglyceride accumulation in the hepatocytes [21]. To determine the effect of HZ extract on esterification in human liver cells and deposition of fatty acid as lipid droplets, HuS-E/2 immortalized human primary hepatocytes were used as a human fatty liver cell model [16]. HuS-E/2 cells were incubated with palmitate and 100 μg/ml of HZ extract for 18 h. The lipid content of the cells was observed by Oil-Red O staining and quantified. As shown in Fig. 2a, compared to HuS-E/2 cells with only palmitate, cells incubated with the HZ extract showed significantly less lipid accumulation. The reduction of cellular lipid accumulation to 39% with the treatment with HZ extract was confirmed by image quantification (Fig. 2b). Because we found HZ extract had an inhibitory effect on lipid deposition in human hepatocytes, possible molecular mechanisms were explored. AMP-activated protein kinase (AMPK) was reported to regulate fat metabolism in the liver and change with cellular energy status [22]. To determine whether HZ extract increased levels of AMPK and its activation, HuS-E/2 cells were incubated with 0.1 mM palmitate in the presence or absence of HZ extract and the expression of AMPK was assessed by western blotting. Levels of phospho-AMPK (pAMPK) at Thr-172 were assessed to evaluate AMPK activation. Ugonins J and K are structurally related to natural flavonoid luteolin [23], which has been demonstrated to attenuate hepatic steatosis [24]. Therefore, the agent luteolin was used as a positive control drug in the subsequent experiment. As shown in Fig. 2c, HZ extract increased AMPK phosphorylation at Thr-172 in palmitate-treated HuS-E/2 cells. In addition, the activation of AMPK’s downstream target enzyme, acetyl-CoA carboxylase (ACC), by phosphorylation at Ser-79 was also measured. HZ extract significantly increased ACC protein phosphorylation. The results indicated that HZ extract facilitated AMPK and ACC activation in HuS-E/2 cells under high-fat conditions. HZ extract showed a stronger effect on AMPK and ACC activation than luteolin in HuS-E/2 cells.

We also identified the changes in proteins associated with fatty acid synthesis and β oxidation. Compared to the palmitate group, HZ extract substantially decreased sterol regulatory element-binding transcription factor 1c (SREBP-1c) protein expression, which is involved in fatty acid synthesis (Fig. 2d). Carnitine palmitoyltransferase I (CPT1) functions as catalyzing fatty acids by β oxidation [25]. Treatment with HZ extract greatly increased CPT1 protein expression, compared to the palmitate group. We determined whether lipid metabolism-related genes in the hepatocytes were influenced by HZ intervention. *The expression of transcription factors,* peroxisome proliferator-activated receptor *alpha (PPARα) and* peroxisome proliferator-activated receptor delta (PPARδ)*, associated with fatty acid β oxidation* were markedly increased by treatment with HZ extract, compared to the palmitate and luteolin groups *(*Fig. 2e*).* The activities of genes involved in de novo lipogenesis in the hepatocytes, *SREBP-1c and peroxisome proliferator-activated receptor gamma (PPARγ) were substantially higher in the* palmitate *group than the non-treated group, while all the genes were expressed at greatly lower levels after treatment with HZ extract and luteolin, compared with the* palmitate *group (*Fig. 2f*).* Taken together, the results suggest HZ extract had a better effect than luteolin on inhibition of fatty acid synthesis and activation of fatty acid β oxidation in palmitate-treated HuS-E/2 hepatocytes.

### HZ lowered the body weight and food efficiency ratio of HFD mice

**Fig. 3:**
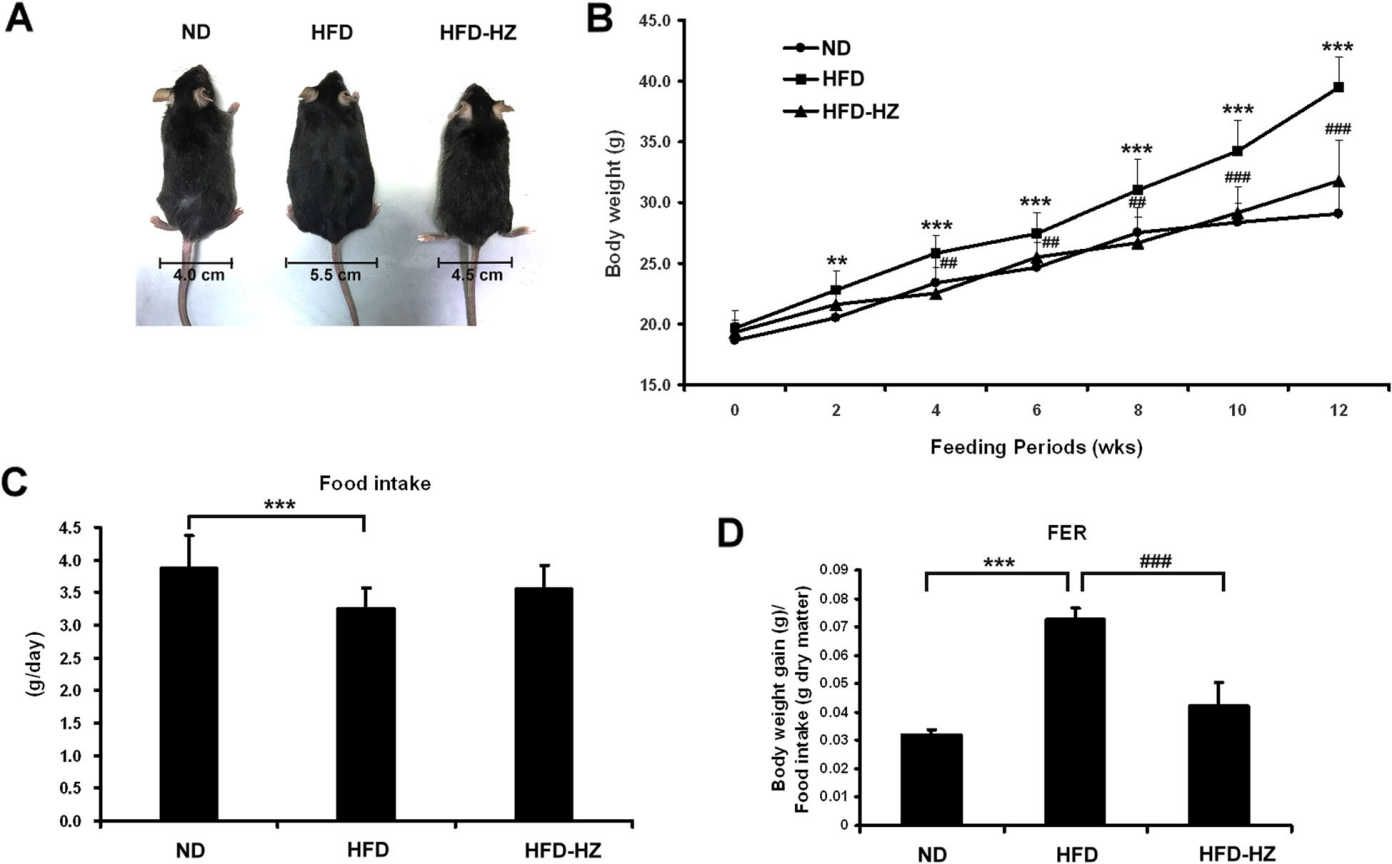
The effect of HZ extract treatment on body weight and food intake in C57BL/6 J mice fed an HFD. **a** Changes in body shape and the waistline. **b** Changes in body weight. **c** Food intake. **d** Food efficiency ratio (Body weight gain/Food intake, FER). Data are shown as means ± SEM (*n* = 10 per group). ND vs. HFD: *p < 0.05; **p < 0.01; ***p < 0.001. HFD vs. HZ: #*p* < 0.05; ##*p* < 0.01; ###*p* < 0.001

The effect of HZ extract on lipemia syndrome and fatty liver was examined in an HFD mouse model. Five-week-old male C57BL/6 J mice were fed with normal diet (ND group, *n* = 10), HFD (HFD group, n = 10), or HFD along with 0.5% lyophilized HZ extract (HFD-HZ group, *n* = 10) for 12 weeks. The morphology of the ND, HFD, and HFD-HZ mice was observed, as shown in Fig. 3a. The size and waist were obviously smaller in the ND group and HFD-HZ group than the HFD mice. The weight of HFD-HZ mice was significantly lower than the HFD mice after 12 weeks of diet supplemented with HZ (Fig. 3b). The food efficiency ratio (FER) was much lower in the HFD-HZ group than the HFD group (Fig. 3d), although the amount of food consumed did not differ significantly (Fig. 3c). This suggests that HZ extract causes reduced food uptake, which may be the reason the mice gain less weight.

### HZ decreased fat deposition in adipocytes

**Fig. 4:**
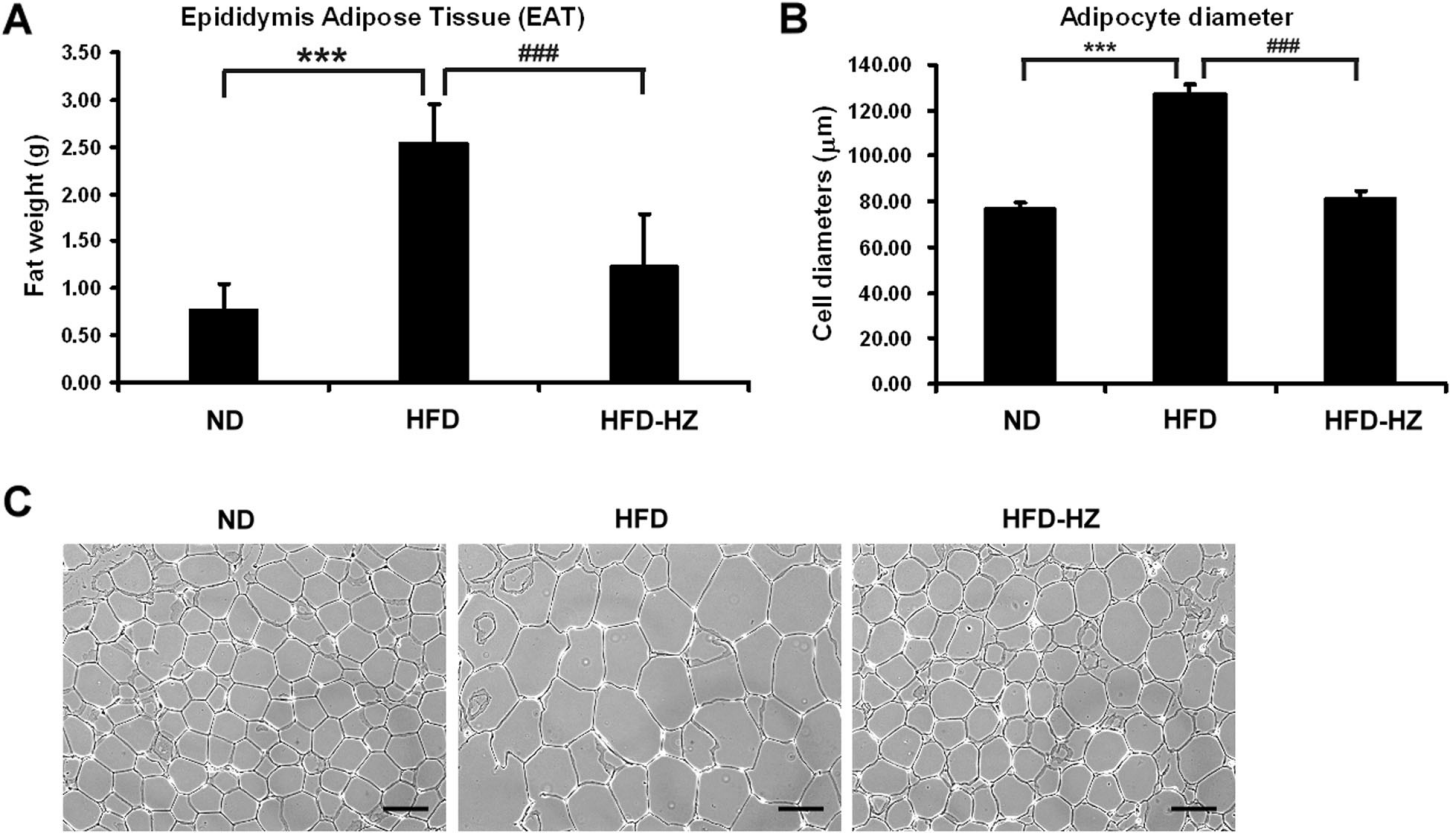
The effect of HZ extract treatment on epididymis adipose tissue (EAT) in C57BL/6 J mice fed an HFD. **a** The weight of EAT. **b** The diameters of adipocytes. **c** hematoxylin-eosin staining of adipocytes in the EAT of mice. The scale bar is 100 μM. Data are shown as means ± SEM (n = 10 per group). ND vs. HFD: *p < 0.05; **p < 0.01; ***p < 0.001. HFD vs. HZ: #*p* < 0.05; ##*p* < 0.01; ###*p* < 0.001

A feature of obesity is increasing lipid accumulation within adipocytes, leading to excessive visceral fat deposits. Therefore, the epididymis adipose tissue (EAT) was dissected and measured after 12 weeks of experimental diet. The mass of EAT in the HFD group was significantly higher than the ND and HFD-HZ groups (Fig. 4a). The adipocytes from the mice that had an HFD with HZ supplement were lower in diameter than those of the HFD group (Fig. 4b and c), which suggests that HZ extract lowers lipid deposition in the mice.

### Hyperlipidemia was prevented after HZ treatment

**Fig. 5:**
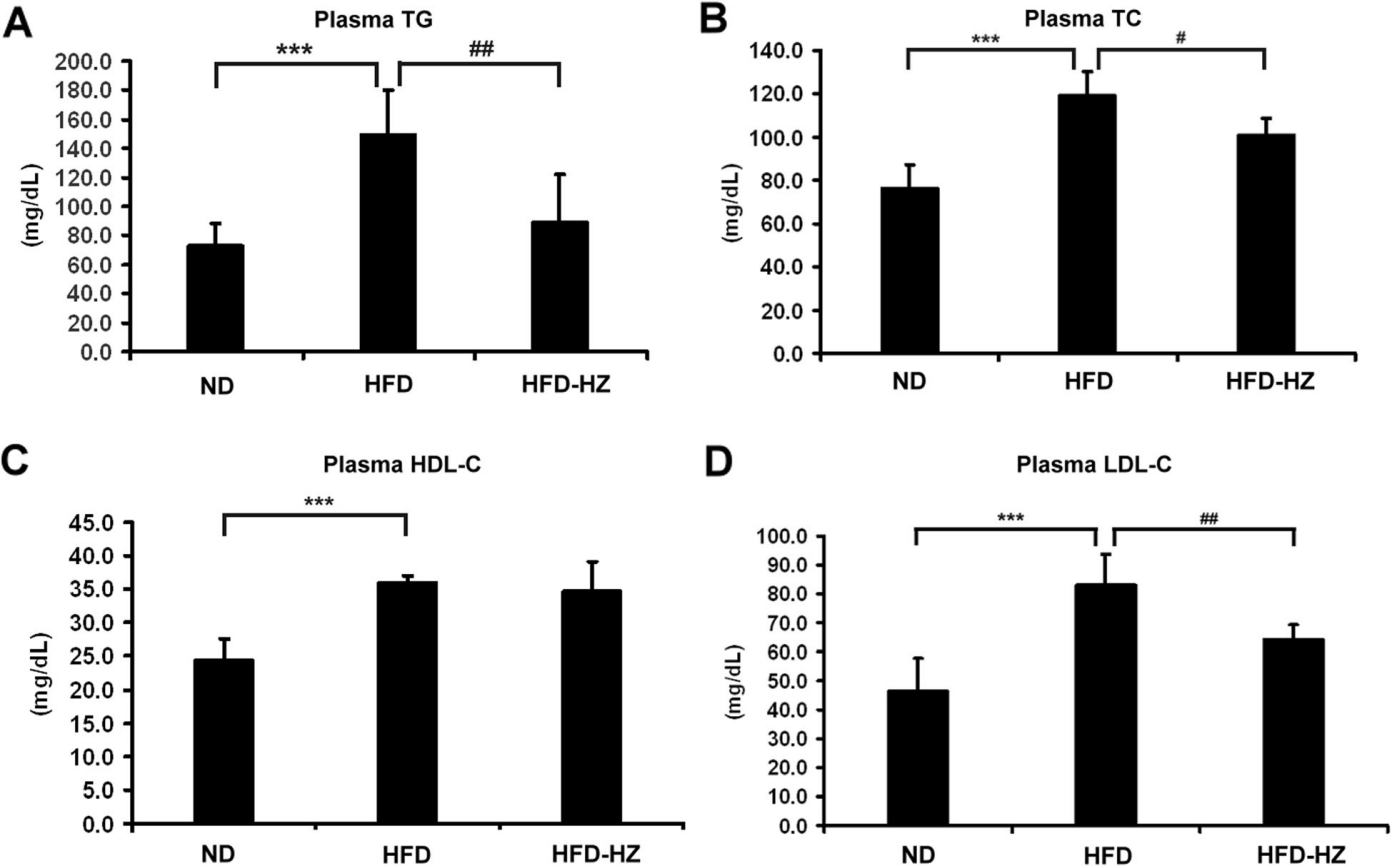
The effect of HZ extract treatment on plasma lipid levels in C57BL/6 J mice fed an HFD. The levels of plasma TG (**a**), TC (**b**), HDL-C (**c**), and LDL-C (**d**). Data are shown as means ± SEM (n = 10 per group). ND vs. HFD: *p < 0.05; **p < 0.01; ***p < 0.001. HFD vs. HZ: #*p* < 0.05; ##*p* < 0.01; ###*p* < 0.001

Alteration of the lipid composition of serum is one of the signs of metabolic problems [26] and plasma lipid levels were monitored after the diet to evaluate the degree of metabolic deficiency. TG, TC, HDL-C, and LDL-C were measured. Significantly higher levels of TG, TC, and LDL-C were expressed in the HFD group than the ND group (Fig. 5a, b, and d). Interestingly, the plasma TG, TC, and LDL-C levels in HFD-HZ group were significantly lower than the HFD group. A high HDL-C level was detected in both the HFD and HFD-HZ groups and may be a consequence of the high cholesterol diet. It is suggested that the presence of hypertriglyceridemia and the high cholesterol phenomenon in the HFD mouse model is consistent with the symptom of obesity in humans, which suggests the HZ extract has the potential to inhibit hyperlipidemia bioactivity.

### The incidence of steatosis under high-fat conditions was lowered by HZ

**Fig. 6:**
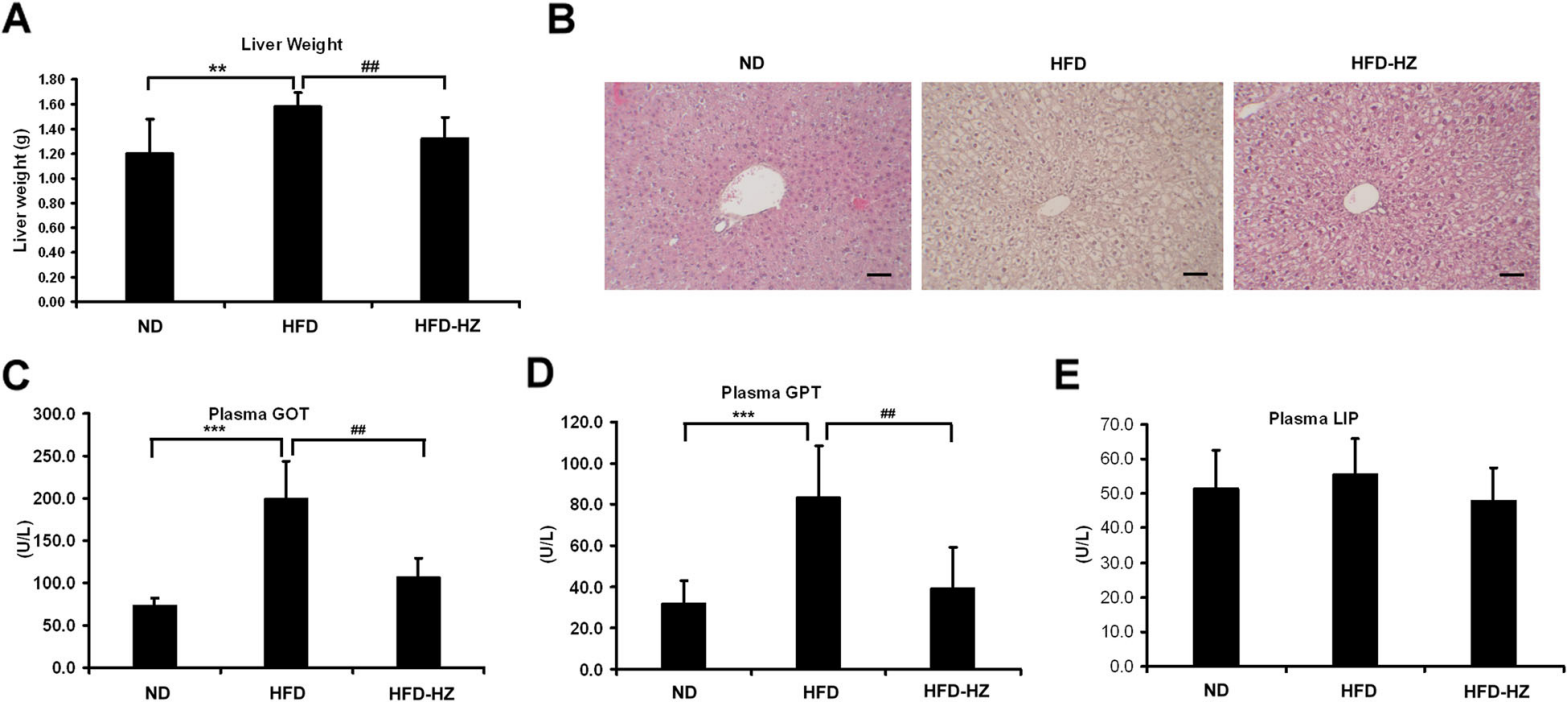
The effect of HZ extract treatment on hepatic steatosis-related markers in HFD-fed C57BL/6 J mice. **a** Changes in liver weight. **b** Hematoxylin and eosin staining of the transverse liver (original magnification × 200). The scale bar is 100 μM. **c** and **d** The plasma levels of the hepatic lipotoxicity markers GOT and GPT. **e** The levels of plasma lipase (LIP). Data are shown as means ± SEM (n = 10 per group). ND vs. HFD: *p < 0.05; **p < 0.01; ***p < 0.001. HFD vs. HZ: #*p* < 0.05; ##*p* < 0.01; ###*p* < 0.001

Non-alcoholic fatty liver disease (NAFLD) is one of the criteria for the development of metabolic syndrome, which is mainly due to triglyceride accumulation in the hepatocytes [27, 28]. To examine the effect of HZ on fatty liver, the liver was weighed and markers of hepatic-steatosis were evaluated to determine the incidence of steatohepatitis. The livers in the HFD group were heavier than the ND group, but lower when the mice had an HFD diet with the HZ extract (Fig. 6a). The increasing weight of the liver may be attributable to lipid accumulation. In addition, the hepatocytes of the HFD mice became swollen with foaming morphology, showing a lack of staining by H&E and suggesting more lipid deposition (Fig. 6b). The cell morphology with H&E staining in the HFD-HZ mouse model was more similar to the ND group. The levels of plasma GOT and GPT help diagnose injury to hepatic tissue [29, 30]. These markers of hepatic injury, GOT and GPT, were upregulated in HFD mice but maintained at lower levels by treatment with the HZ extract (Fig. 6c and d). There were no significant effects on the pancreas inflammatory marker, LIP (Fig. 6e). These data suggest that HZ extract specifically prevented diet-induced hepatic steatosis and inflammatory response in the liver.

### HZ rescued HFD-induced insulin resistance syndrome

**Fig. 7:**
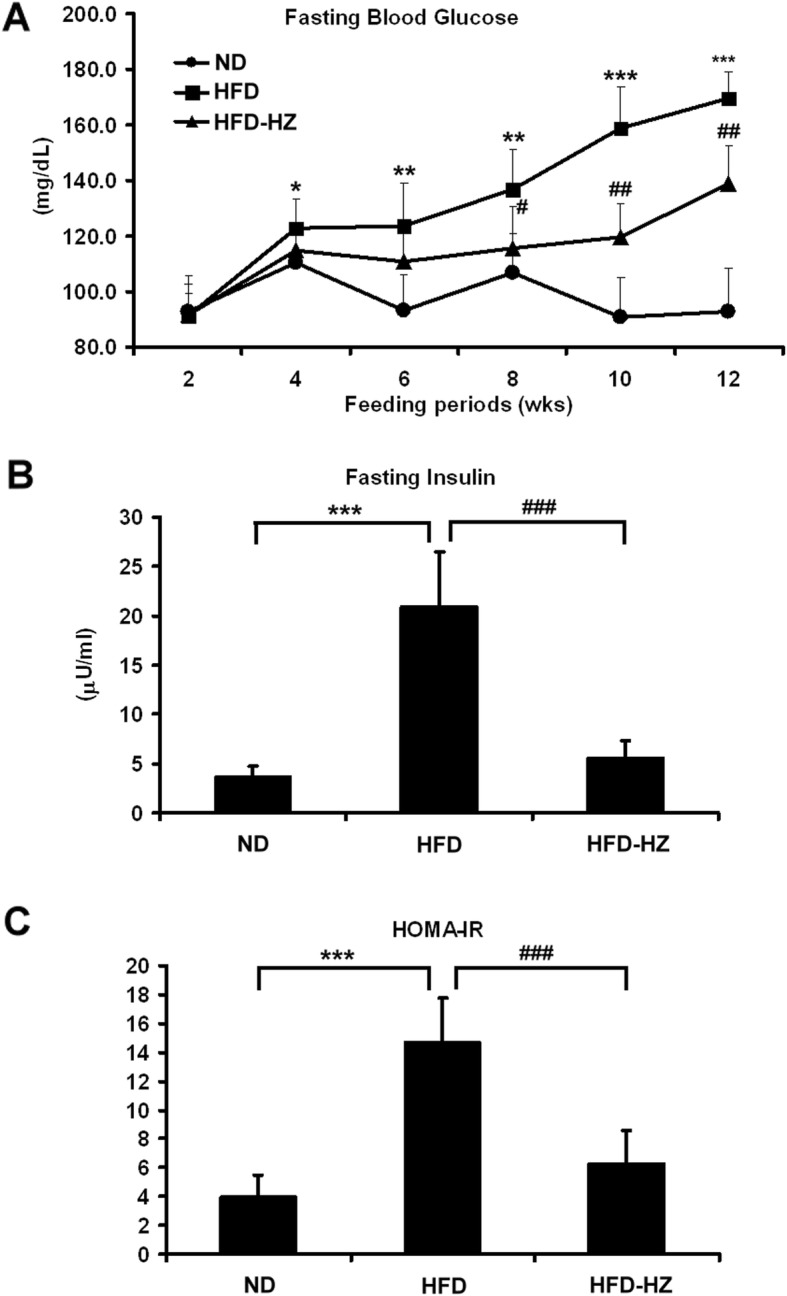
The effect of HZ extract treatment on blood glucose and insulin resistance in HFD-fed C57BL/6 J mice. **a** Levels of blood glucose after 12 h of fasting. **b** Levels of plasma insulin after 12 h of fasting. **c** The HOMA-IR index calculated using fasting blood glucose and insulin levels. Data are shown as means ± SEM (n = 10 per group). ND vs. HFD: *p < 0.05; **p < 0.01; ***p < 0.001. HFD vs. HZ: #*p* < 0.05; ##*p* < 0.01; ###*p* < 0.001

Previous studies have confirmed the association between dietary fat intake and deteriorated insulin function [31]. The fasting-glucose and insulin levels in HFD mice with or without HFD or HZ extract were measured, reflecting the incidence of insulin resistance syndrome. High fasting blood glucose levels in the HFD group suggested an abnormality of insulin function, while this was prevented by HZ extract treatment (Fig. 7a). The uncontrolled high-fasting insulin level is considered as the earliest sign of the onset of metabolic syndrome [32]. Interestingly, the increase in insulin levels was prevented in the HFD-HZ mice, compared to the HFD group (Fig. 7ba). Generally, insulin resistance is monitored using the homeostasis model assessment of insulin resistance (HOMA-IR) [33]. Therefore, the high HOMA-IR index reflected the increased incidence of diabetes symptoms in HFD mice with high fasting blood glucose and insulin levels. The HOMA-IR index was calculated and found to be at the control level with HZ extract treatment (Fig. 7c), indicating inhibition of insulin resistance.

## Discussion

Metabolic syndrome associated with insulin resistance has been recognized as a prevalent health issue for decades and has become a burden to many health care systems [34]. Moreover, NAFLD is considered to be closely associated with obesity and metabolic syndrome [35]. Herbal medicines have been shown to have potential therapeutic effects in regulating blood glucose, blood lipid and weight, which are indices for evaluating metabolic syndrome [36]. In our study, HZ extract was prepared, analyzed and its bioactivity examined in regulating an HFD-induced metabolic disorder. The major components of HZ extract were found to be ugonin J and ugonin K (Fig. 1). Consistent with previous findings, HZ extract was predominantly composed of cyclized geranylflavonoids [37]. Our results showed that when HFD mice were treated with an HZ extract, they were protected against HFD-induced metabolic syndrome.

AMPK is well known to be one of the proteins that regulate metabolic pathways [38]. AMPK activation may protect the liver from lipid accumulation, insulin resistance, and glucose tolerance induced by HFD [39]. Previous studies also showed hepatic activation through the phosphorylation of AMPK suppresses fatty acid synthesis [40]. Our in vitro study showed that HZ extract significantly increased the phosphorylation of AMPK and the phosphorylation of AMPK’s downstream enzyme ACC. This suggests HZ extract facilitated AMPK and ACC activation in cells under high-fat conditions, and reduced palmitate-induced cellular lipid accumulation in hepatocytes.

Generally, an HFD promotes severe changes, such as hepatic steatosis, β oxidation status and the balance of oxidants, which has effects on body weight, insulin signaling and other metabolic parameters [41]. HFD mice have abnormalities in lipid and glucose metabolism. Here, with the human fatty liver cell model and the HFD mice model, we aimed to demonstrate the effects of the HZ extract on the pathogenesis of liver-related metabolic syndrome and dyslipidemia. In terms of protecting the liver from oxidative stress and inflammation, we confirmed the effects of HZ extract on regulating lipid metabolism. However, the pharmacokinetics of HZ extract remains unclear and needs further clarification.

Previously, twelve flavonoids were identified in ethanol-extracted HZ through HPLC and NMR analysis [11]. Among the flavonoids in HZ, many bioactivities have been demonstrated, such as antioxidative activity [11], anti-inflammatory function [37], antiosteoporosis [12], and anti-cancer [42, 43] and hepatoprotective effects [44]. In addition, plant-derived flavondoids recently were proposed as a health supplement [45]. For example, tea-extracted catechins and theaflavins improved plasma lipid absorption [46] and have been used for hyperlipidemia therapy. Furthermore, solid evidence proved that cinnamon extract regulated the metabolism of carbohydrates and lipids through peroxisome proliferator-activated receptors (PPARs) in obesity and diabetic mouse models [47, 48]. Moreover, anthocyanin C3G, a flavonoid, inhibited excessive ROS production by activating GSH synthesis, which many consider to be controllers of hyperglycemia-induced hepatic oxidative damage [49]. Based on this evidence of the therapeutic potential of flavonoids, the HZ extract was shown to be effective in restoring metabolic syndrome induced by an HFD. However, the detailed mechanisms of action of ugonins J and K, and perhaps other bioactive compounds in HZ, require to be elucidated.

## Conclusions

Our results showed that HZ extract prevented increases in body weight and fat accumulation around the waist in mice fed a 12-week-HFD diet. Food intake efficiency was decreased along, with a reduction in the accumulation of fat that may cause metabolic syndrome. HZ treatment also inhibited the extremely high level of bad cholesterol transportation carriers, LDL-C, in HFD mice, these being considered a sign of health problems. The protective effect on metabolism was examined in terms of concentrations of glucose, lipids, and other insulin resistance-associated index. Further investigation of the molecular mechanism showed that HZ extract upregulated the genes and proteins associated with fatty acids oxidation, downregulated those related to hepatic de novo lipogenesis in palmitate-treated human HuS-E/2 hepatocytes. These solid results indicate that HZ has a promising bioactivity in regulating obesity and insulin sensitivity, which may have potential for clinical application in preventing from hepatic steatosis and insulin resistance.

## Supplementary information

**Additional file 1: Table S1.** Primer sequences in RT-PCR

## Data Availability

The datasets used and/or analyzed during the current study available from the corresponding author on reasonable request.

## Abbreviations

ACC: Acetyl-CoA carboxylase
AMPK: AMP-activated protein kinase
CPT1: Carnitine palmitoyltransferase I
EAT: Epididymis adipose tissue
FER: Food efficiency ratios
GOT: Glutamic oxaloacetic transaminase
GPT: Glutamic pyruvic transaminase
HDL-C: HDL-cholesterol
HFD: High fat diet
HOMA-IR: Homeostasis model assessment of insulin resistance
HZ: *Helminthostachys zeylanica*
LDL-C: Low-density lipoprotein cholesterol
LIP: Lipase
NAFLD: Nonalcoholic fatty liver disease
ND: normal diet
*PPARα*: Peroxisome proliferator-activated receptor *alpha*
*PPARγ*: *Peroxisome proliferator-activated receptor gamma*
PPARδ: Peroxisome proliferator-activated receptor delta
SREBP-1c: Sterol regulatory element-binding transcription factor 1c
T2DM: Type 2 diabetes mellitus
TC: Total cholesterol
TG: Triglyceride
