# Supplementary figures and images for: Helminthostachys zeylanica alleviates hepatic steatosis and insulin resistance in diet-induced obese mice
Source: BMC Complement Altern Med. 2019 Dec 13;19:368. doi: 10.1186/s12906-019-2782-3 (PMC6911300; doi:10.1186/s12906-019-2782-3)

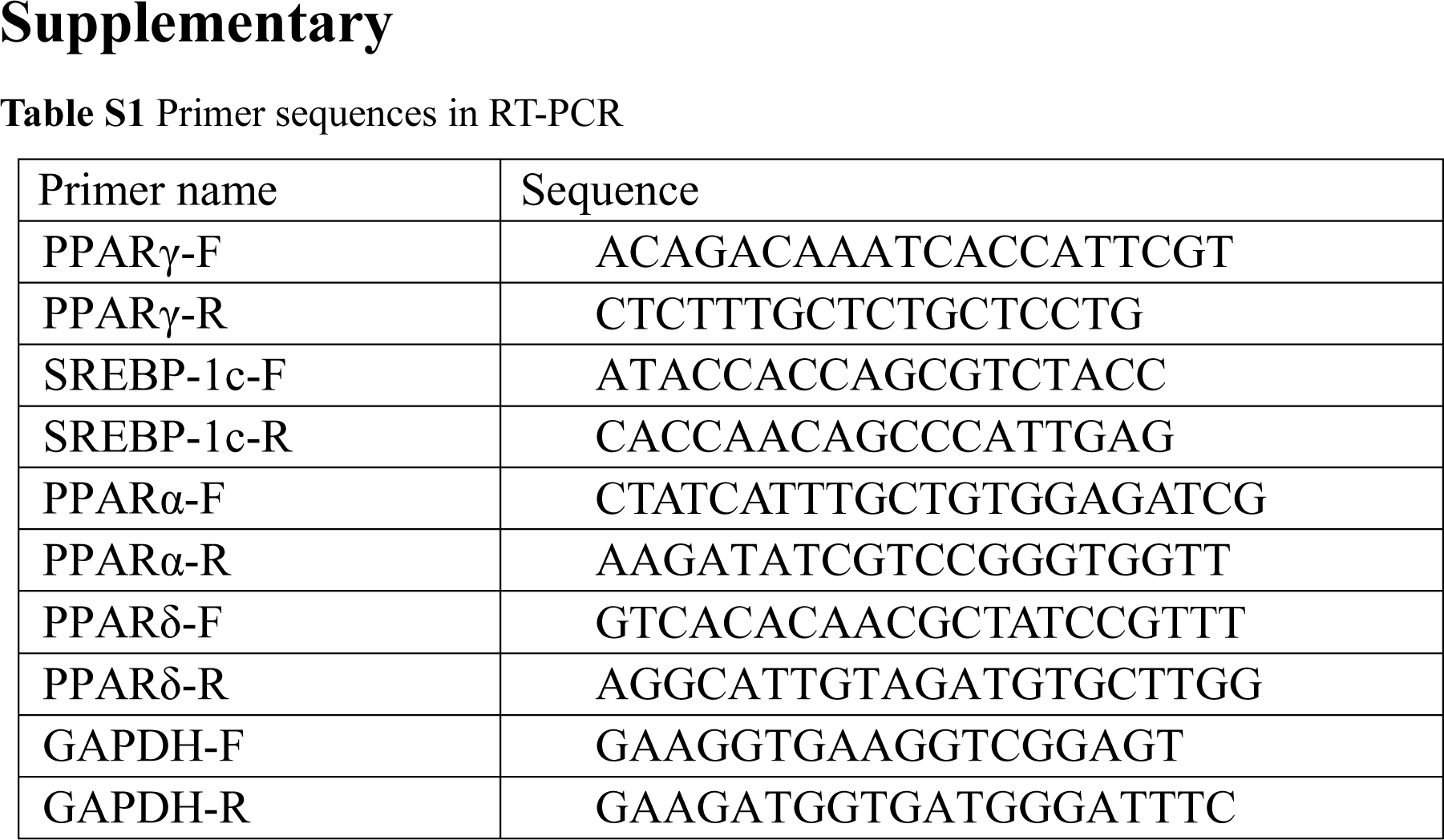

Supplement: Supplementary file 1 — Additional file 1: Table S1. Primer sequences in RT-PCR [file 12906_2019_2782_MOESM1_ESM.tif]
